# Protein Kinase A Signaling Inhibits Iridophore Differentiation in Zebrafish

**DOI:** 10.3390/jdb6040023

**Published:** 2018-09-26

**Authors:** Cynthia D. Cooper, Steve D. Erickson, Scott Yin, Trevor Moravec, Brian Peh, Kevin Curran

**Affiliations:** 1 School of Molecular Biosciences, Washington State University Vancouver, Vancouver, WA 98686, USA; 2 College of Arts and Sciences, Washington State University Vancouver, Vancouver, WA 98686, USA; stevederickson@gmail.com (S.D.E.); scottyin@yahoo.com (S.Y.); moravect@wsu.edu (T.M.); brianpeh@comcast.net (B.P.); 3 Department of Biology, University of San Diego, San Diego, CA 92110, USA; kevincurranuw@gmail.com

**Keywords:** iridophores, PKA signaling, forskolin, melanophores, differentiation, *mitfa*, *pnp4a*

## Abstract

In zebrafish (*Danio rerio*), iridophores are specified from neural crest cells and represent a tractable system for examining mechanisms of cell fate and differentiation. Using this system, we have investigated the role of cAMP protein kinase A (PKA) signaling in pigment cell differentiation. Activation of PKA with the adenylyl cyclase activator forskolin reduces the number of differentiated iridophores in wildtype larvae, with insignificant changes to melanophore number. Inhibition of PKA with H89 significantly increases iridophore number, supporting a specific role for PKA during iridophore development. To determine the effects of altering PKA activity on iridophore and melanophore gene expression, we examined expression of iridophore marker *pnp4a*, melanophore marker *mitfa*, and the *mitfa* repressor *foxd3*. Consistent with our cell counts, forskolin significantly decreased *pnp4a* expression as detected by in situ hybridization and quantification of *pnp4a+* cells. Forskolin had the opposite effect on *mitfa* and *foxd3* gene activity, increasing the area of expression. As *mitfa/nacre* mutants have extra iridophores as compared to wildtype larvae, we examined the function of *mitfa* during PKA-sensitive iridophore development. Forskolin treatment of *mitfa/nacre* mutants did significantly reduce the number of iridophores but to a lesser extent than that observed in treated wildtype larvae. Taken together, our data suggests that PKA inhibits iridophore development in a subset of iridophore precursors, potentially via a *foxd3*-independent pathway.

## 1. Introduction

Chromatophores, or pigment/light-reflecting cells, develop from neural crest cells, a population of cells that arise near the dorsal aspect of the developing vertebrate neural tube. Five types of chromatophores have been characterized in different organisms, including several species of fish. These cell types include black melanophores, yellow xanthophores, silver iridescent iridophores, white reflective leucophores, and red erythrophores (reviewed in [1]). Chromatophores promote temporary or permanent color change and are thus critical for an aquatic organism’s ability to evade predators, to blend into their environments, or to find mates. Zebrafish have a subset of these chromatophores, including melanophores, xanthophores, and iridophores. Each cell type represents an excellent system for understanding cell fate decisions and subsequent differentiation. Melanophores are the best characterized and loss of function mutations in zebrafish microthalmia-associated transcription factor (*mitfa)*, *kit*, oculocutaneous albinism 2 (*oca2*), and vacuolar protein sorting 11 (*vps*) genes promote phenotypes similar to those found in mammalian knockouts (i.e., loss of pigment cell precursors or under/hypopigmented melanocytes; [2,3,4,5,6,7,8,9]). Some pathways affecting melanocyte development also impact iridophore development, offering a separate system for understanding the activity or function of these pathways during cell differentiation. For example, the number of iridophores is impacted by several loss of function mutations in genes previously characterized in melanocytes, including *oca2* (increased iridophores), *vps11* (decreased iridophores), and *mitfa* (increased iridophores) as compared to age-matched wildtype larvae. Additionally, Forkhead transcription factor *foxd3* regulates the expression of *mitfa* and nucleoside phosphorylase gene, *pnp4a,* in a bipotent melanophore/iridophore precursor cell, suggesting that a population of cells exists with the capability of becoming either pigment cell type [10]. It remains unclear as to which intracellular signaling proteins control iridophore differentiation decisions. Here, we propose a role for protein kinase A (PKA) in inhibiting the iridophore differentiation program.

PKA signaling is tightly controlled by regulation of cAMP gradients. Adenylyl cyclases generate cAMP and phosphodiesterases—the latter degrades cAMP (reviewed in [11]). Upon activation by cAMP (via regulatory unit binding), protein kinase A catalytic units phosphorylate target proteins involved in several cellular processes, including cell differentiation and growth [12,13,14,15]. PKA signaling also functions in organelle trafficking within chromatophores. Melanophore melanin-producing organelles, melanosomes, are trafficked towards the cell periphery (dispersion) or nucleus (aggregation) in response to hormones such as cAMP-dependent melanocyte-stimulating and melanocyte-concentrating hormones, respectively [16]. Similarly, xanthophore pigment-producing organelles, xanthosomes, internalize their pigment granules in response to changes in light in a cAMP- and phosphodiesterase-dependent manner [17]. Last, PKA signaling also regulates transcriptional programming important for neural crest cell fate choice in quail cell cultures, resulting in the induction of the melanocyte differentiation gene *Mitf* while simultaneously inducing expression of the RE-1 silencing transcription factor, a neuron-specific transcriptional repressor. These results were duplicated in zebrafish models where involvement of the Wnt signaling responsive transcription factor, CtBP2, was required for increased melanophore differentiation [18], suggesting a cross-talk between Wnt and PKA signaling. Thus, there is previous evidence of PKA serving as a switch to encourage differentiation of one lineage at the expense of a second. We explored a function for PKA signaling during iridophore development. Our data suggests that PKA signaling inhibits iridophore differentiation while promoting differentiation of melanophores in zebrafish larvae.

## 2. Materials and Methods

### 2.1. Fish Rearing and Crosses

Zebrafish work is approved by Washington State University Institutional Animal Care and Use Committee, Animal Subject Approval Form 03848. Wildtype fish were of the AB (ZDBGENO-960809-7) strain. Adult zebrafish were maintained on a 14/10 h light/dark cycle at 28.5 °C. Adult fish were maintained on a recirculating water system, fed dried/live diets, and checked daily. Embryos were acquired from natural crosses and grown at 28.5 °C in embryo media until analysis. Embryos were staged according to characterized morphological criteria [19]. The following mutant alleles were used: *nacre* [7]. 

### 2.2. Production of BACfoxd3::GFP Transgenic Zebrafish

We modified a *foxd3*-containing bacterial artificial chromosome (BAC) clone by *Escherichia coli*-based homologous recombination [20]. BAC clone 137512 contains around 130 kb of sequence upstream and 40 kb downstream of *foxd3* (http://www.sanger.ac.uk/Projects/D_rerio/mapping.shtml). BAC clone 137512 was inserted into pTAR BAC 2.1. Following recombination, the modified BAC clone contained an *egfp* gene positioned at an endogenous start site. The accuracy of recombination was evaluated by PCR, sequencing, and by transient expression assays. *BACfoxd3::*EGFP faithfully recapitulated endogenous *foxd3* expression in neural crest and other organs. To obtain a germline, we linearized BAC DNA with *Not 1*, injected linearized BAC DNA into zebrafish embryos, raised injected fish to adulthood, and screened their progeny for reporter gene expression. The germline transmission rate was 2%. 

### 2.3. Forskolin and H-89 Treatments

Stocks of forskolin and H-89 were prepared in dimethyl sulfoxide (DMSO) at concentrations of 2.4 and 9.6 mM, respectively, and stored at −20 °C until use. Zebrafish were treated with 5 µM forskolin (Sigma, St. Louis, MO, USA) diluted in embryo media beginning at 17–20 h post fertilization (hpf) unless otherwise indicated. At 48–50 hpf, forskolin-containing medium was removed, and fish were rinsed twice in fresh embryo media and incubated at 28.5 °C until fixation. At the indicated timepoint, fish were fixed in 4% paraformaldehyde (4% PFA, diluted in phosphate-buffered saline, PBS), rinsed in PBS +0.01% Tween (PBTw), and stored in PBTw (pre-in situ) until in situ hybridization/quantification or PBS +0.01% Triton X-100 (PBTx) until antibody staining. Samples were stored in 50% glycerol/PBS for analysis and to preserve fluorescence of antibody-stained samples. For H-89 (Sigma, St Louis, MO, USA) treatment, fish were treated with 25 µM or 50 µM H-89 in embryo media beginning at 17–20 hpf unless otherwise indicated. AT 48–50 hpf, H-89-containing media was removed, and fish were rinsed twice in fresh embryo media and incubated at 28.5 °C until fixation for quantification. H-89 treated fish were fixed using 4% PFA and stored in PBTw until in situ hybridization analysis or 50% glycerol/PBS until quantification. 

### 2.4. Cell Quantification and Statistics

For iridophore quantification, a fiber optic light source was used to illuminate iridophores. Iridophores were counted using Nikon SMZ-1500 (Nikon Inc., Melville, NY, USA) or Leica M80 (Leica Microsystems, Inc., Buffalo Grove, IL, USA) stereomicroscopes. Unless otherwise indicated, cell counts include all (dorsal and ventral) trunk and tail iridophores. Fixed fish were used for quantification and 6% methyl cellulose was used to position fish for counting. Fish were rotated in methyl cellulose as necessary for viewing individual stripes/locations. For in situ hybridization analysis, fish were divided into “anterior body”, “posterior body”, and “tail” regions (see Figure D’ for additional information). Anterior regions include tissue dorsal to rostral and caudal yolk boundaries, while posterior regions include tissue posterior to the caudal yolk boundary, ending at the end of the yolk extension. The tail region included tissue posterior to the yolk extension. Statistical tests performed for each experiment were the Student’s *t*-test (Microsoft Excel 2007) or ANOVA (one- or two-way ANOVA; GraphPad Prism, San Diego, CA, USA). Data reported include a minimum of 8–10 samples per time point, with at least three replicates per experiment.

### 2.5. In Situ Hybridization

Embryos were fixed in 4% paraformaldehyde in phosphate-buffered saline at pH 7.2, and processed using standard protocols [21]. Digoxigenin-labeled probes for *mitfa* [7], *foxd3* [22,23], and *pnp4a* [10] have been previously described. All imaging was done using a Nikon SMZ-1500 stereomicroscope equipped with a Digital Sight DS-Ri1 Digital Camera. All images were processed for contrast, brightness, and color using Adobe Photoshop CS3 Extended Version 10.0 (Adobe Corporate Headquarters, San Jose, CA, USA). 

### 2.6. Antibody Staining and Confocal Imaging

The following antibodies were used for immunohistochemistry at the indicated dilutions: mouse monoclonal anti-Green Fluorescent Protein (Invitrogen/ThermoFisher Scientific, Waltham, MA, USA), 1:500; rabbit anti-beta catenin (Pharmingen, San Diego, CA, USA), 1:100; anti-mouse (Alexa 488) and anti-rabbit (Alexa 568) secondary antibodies (Molecular Probes, Eugene, OR, USA) 1:500 or 1:750. Brightfield or incident lighting (epi-illumination from a fiber optic/ectopic light source) images were obtained on a Nikon dissecting microscope with a Spot RT Slider digital camera (Diagnostic Instruments, Grand Island, NY, USA) or a Nikon SMZ-1500 stereomicroscope with a Digital Sight DS-Ri1 Digital camera. Fluorescent confocal images were obtained on LSM 5 Pascal (Zeiss, Thornwood, NY, USA) or Olympus FV1000 laser scanning confocal microscopes. Images were equivalently processed for color balancing and brightness/contrast using Photoshop CS3 or CS4 (Adobe, San Jose, CA, USA) and formatted with Illustrator CS4 (Adobe, San Jose, CA, USA). 

## 3. Results and Discussion

### 3.1. Effects of Protein Kinase A Activity Alter the Number of Differentiated Iridophores

**Figure 1:**
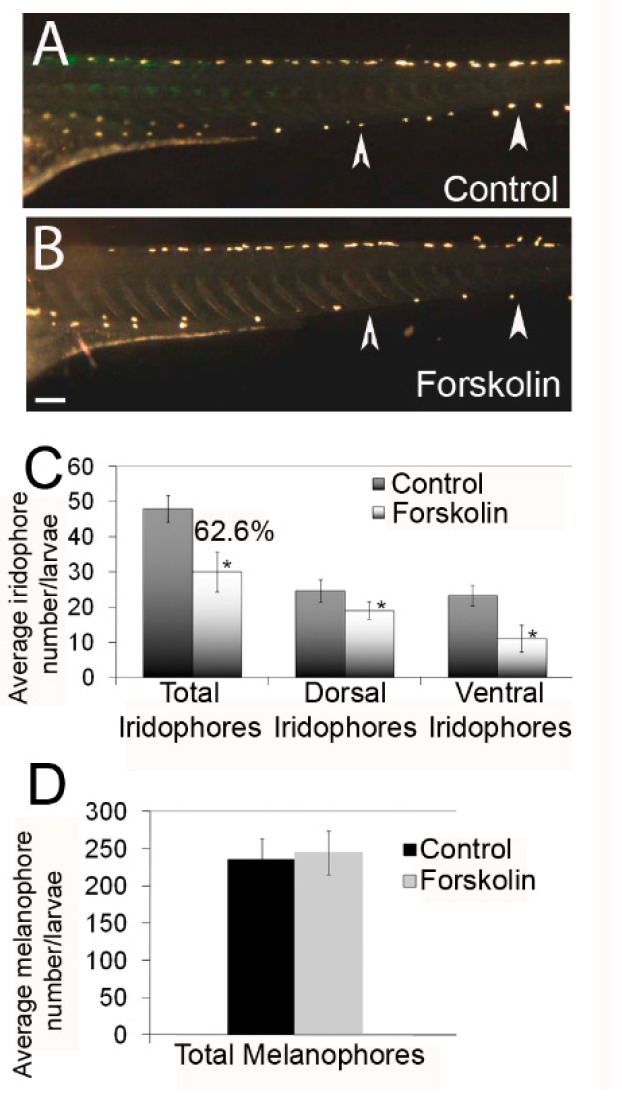
Forskolin treatment reduces iridophore number in zebrafish larvae at 4 days post fertilization (dpf). (**A**,**B**) Lateral images (using an incident lighting source) of larvae at 4 dpf treated with 0.1% dimethyl sulfoxide (DMSO) or 5 µM forskolin in embryo media. Forskolin-treated individuals have fewer iridophores, especially in the ventral stripe (arrowheads). Scale bar = 200 um and applies to panels A and BC) Quantification of total, dorsal stripe, and ventral stripe iridophores (*n* = 8–10 larvae per condition). Forskolin treatment leads to a 38.4% reduction in differentiated iridophores (62.6% of control number). (**D**) Quantification of melanophores following 0.1% DMSO or 5 µM forskolin treatment. Total melanophores include dorsal, lateral, and ventral stripes (*n* = 8–10 larvae per condition), showing no significant change following forskolin treatment.

To examine the role of protein kinase A during iridophore development, we treated zebrafish embryos with forskolin, an adenylyl cyclase activator. Treatments commenced at 17–20 h post fertilization (hpf) when iridophores are specified from neural crest cells [10]. Following washout of forskolin at 2 days post fertilization (dpf), larvae were grown until 4 dpf when they were fixed for iridophore quantification. At this timepoint, iridophores are positioned ~one cell per somite, making them easy to quantify. Using an incident light source, we were able to illuminate and observe individual iridophores in the dorsal and ventral stripes, beginning at the anterior trunk level. Imaging analysis indicates fewer iridophores in forskolin-treated individuals, especially in the ventral stripes (Figure 1A,B; white arrowheads indicate the ventral stripe). Quantification of iridophores in dorsal and ventral stripes confirms a significant reduction in both stripes, and a 38.4% reduction in total iridophores (Figure 1C, *p* < 0.05 via Student’s *t*-test). To ensure this reduction was not due to developmental delay of the larvae, we also quantified melanophores which develop at a similar time to iridophores in zebrafish (Figure 1D). Statistical analysis suggests that while melanophore number is slightly higher with forskolin treatment (as compared to controls), this increase is not significant (Student’s *t*-test, *p* > 0.05; we also noted no significant differences in dorsal or ventral stripe numbers). Normal somite shape and yolk extension patterning/development further suggest minimal impact on other tissues in forskolin-treated larvae.

**Figure 2:**
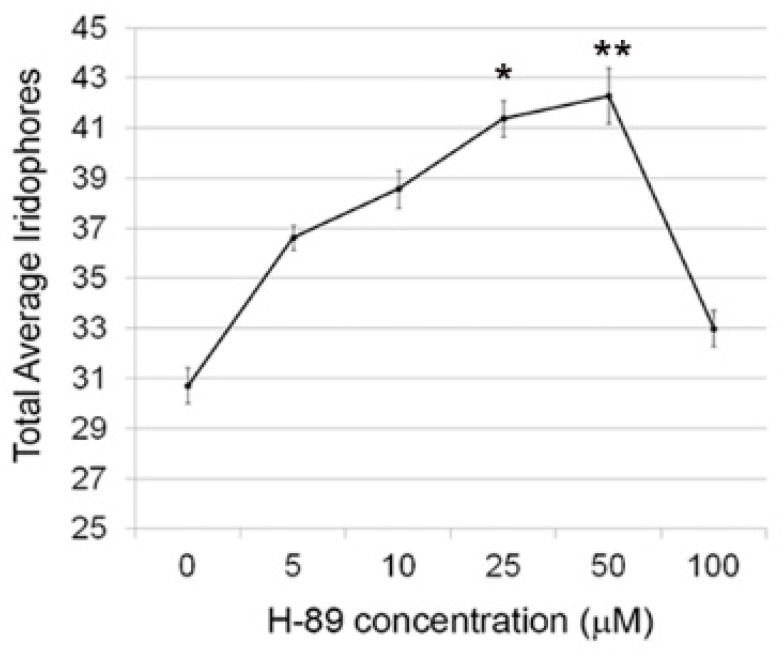
H89 treatment increases iridophore number in zebrafish larvae at 4 dpf. Quantification of iridophores following treatment of wildtype embryos with varying doses of the protein kinase A (PKA) inhibitor H89. One-way ANOVA with Bonferonni multiple comparison post-test indicates that doses of 25 µM and 50 µM are statistically significant (* *p* < 0.05 or ** *p* < 0.01).

To confirm that forskolin effects were specific to protein kinase A, we analyzed the effects of the protein kinase A inhibitor, H-89 dihydrochloride (H-89), on iridophore number. We tested several concentrations to best understand the effects of distinct doses on iridophore development (Figure 2). Inhibition of protein kinase A via 25 µM and 50 µM H-89 treatment significantly increases iridophore number (one-way ANOVA with Bonferroni multiple comparison (* *p* < 0.05 or ** *p* < 0.01)), consistent with our forskolin data. We do see a lower than expected number of iridophores at 100 µM, most likely due to developmental delay or toxicity with this dose. Taken together, this data suggests that protein kinase A functions to regulate the number of differentiated iridophores in developing zebrafish.

### 3.2. PKA Modulation Inhibits Iridophore Gene Expression

**Figure 3:**
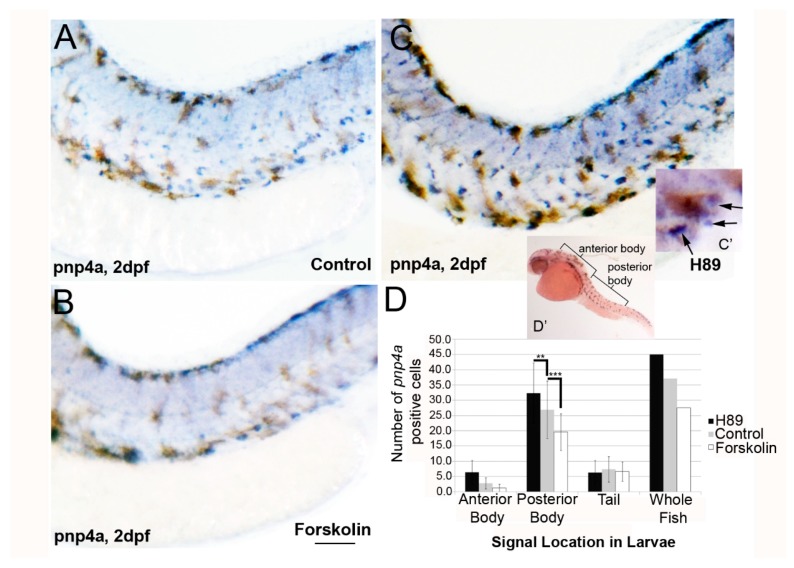
**Forskolin treatment reduces iridoblast marker *pnp4a* expression.** (**A**–**C**) Lateral brightfield images of larvae at 2 dpf processed for in situ hybridization using *pnp4a* probes, following treatment with 0.1% DMSO (**A**), 5 µM forskolin (**B**), or 25 µM H-89 (**C**) in embryo media. Activation of adenylyl cyclase (and PKA signaling) reduces the levels of *pnp4a* expression, whereas inhibition of PKA has the converse effect (increased *pnp4a* expression). Scale bar = 200 um andapplies to all images. (**C’**) Representative image with black arrows showing blue/purple *pnp4+* cells included in panel D quantification. Brown melanophores were not included in counts. (**D**) Quantification of *pnp4a+* cells confirms a significant reduction and increase in the number of *pnp4a+* cells following forskolin and H-89 treatment, respectively (** *p* < 0.01 and *** *p* < 0.0001 via two-way ANOVA and Bonferonni multiple comparison analysis). (**D’**) The quantified regions are labeled.

Zebrafish iridophores are specified and then differentiate beginning at 20 h post fertilization (hpf), as detected by purine nucleoside phosphorylase (*pnp4a*) expression [10]. As PKA signaling functions during neuron differentiation [22], we hypothesized that PKA signaling regulates differentiation of the iridophore precursors, iridoblasts. To test this hypothesis, we examined *pnp4a* expression in forskolin and H89-treated larvae. Embryos were treated with forskolin, H89, or control (0.1% DMSO) embryo medium beginning at 22 hpf. Larvae were fixed at 2 dpf and analyzed via in situ hybridization for *pnp4a* expression. Forskolin treatment reduced the amount of *pnp4a+* signal at 2 dpf (posterior body; Figure 3A,B). Conversely, H89 individuals showed an increase in *pnp4a* signal (compare Figure 3C to Figure 3A). To determine if the number of *pnp4a+* cells had significantly changed, we quantified the number of *pnp4a+* cells in the anterior body, posterior body, and tail. The number of *pnp4a+* cells in posterior body/trunk regions of forskolin-treated larvae is significantly lower as compared to DMSO-treated controls (Figure 3D; *** *p* < 0.0001 via two way ANOVA with Bonferroni multiple comparisons analysis) and significantly higher with H89 treatment (** *p* < 0.01 via two way ANOVA with Bonferroni multiple comparisons analysis) at 2 dpf. This data suggests that PKA activity inhibits iridophore differentiation by reducing *pnp4a* expression.

### 3.3. mitfa Is Partially Required for the Forskolin-Dependent Reduction in Iridophore Number

**Figure 4:**
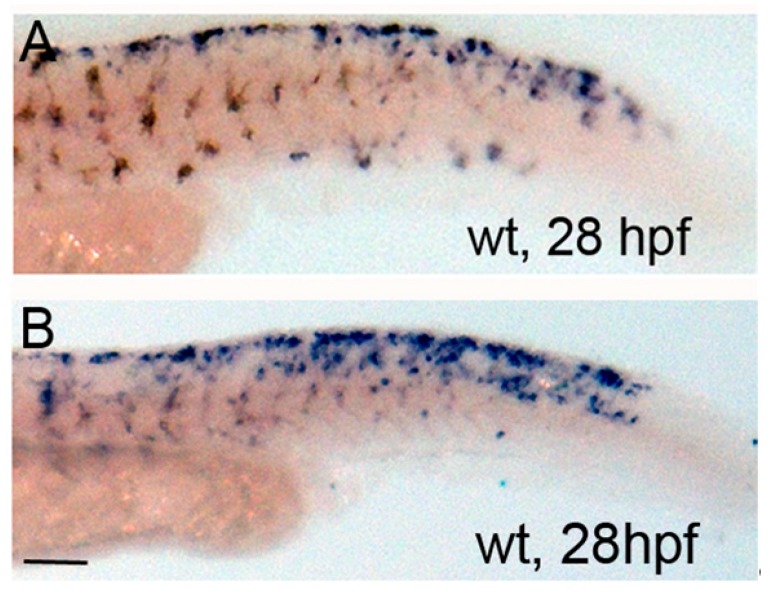
Forskolin treatment expands *mitfa* expression at 28 hpf. (**A**,**B**) Brightfield images of lateral tail regions of 28-h-old wildtype control and forskolin-treated embryos processed for in situ hybridization using *mitfa* probe. Note increased *mitfa* expression in the dorsal tail. Scale bar = 100 um and applies to both images.

*mitfa* is required and sufficient for melanophore specification as indicated by *nacre*/*mitfa* zebrafish mutants, which do not develop melanophores [7]. Additionally, iridophores are present in excess in *mitfa* mutants as compared to wildtype control zebrafish [7,10], suggesting that *mitfa* is critical for establishing correct iridophore number. Examination of simultaneous *pnp4a* and *mitfa* expression indicates co-expression of these gene products at 24 hpf (but loss of co-expression by 50 hpf [10]). To examine the requirement of *mitfa* during PKA-sensitive iridophore differentiation, we first observed *mitfa* expression with or without PKA activation (forskolin treatment). Forskolin-treated larvae showed larger regions of *mitfa* expression posterior to the eye and in the dorsal tail as compared to DMSO treated controls (Figure 4A,B). 

**Figure 5:**
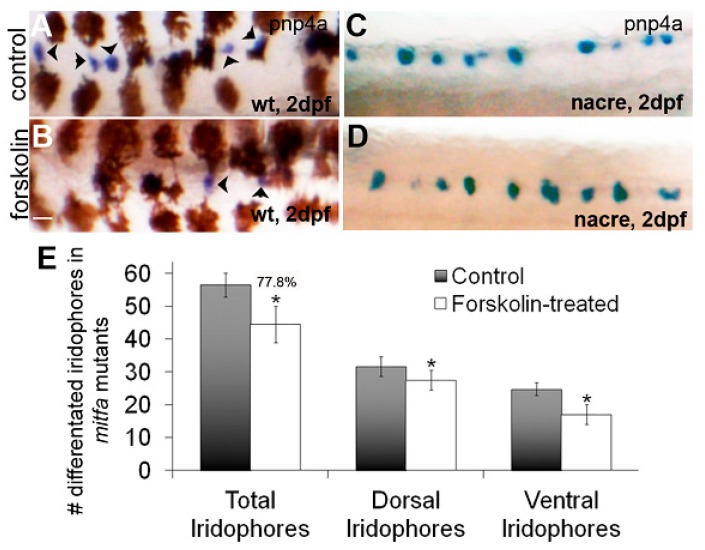
A subset of *mitfa* negative iridoblasts is resistant to PKA signaling. (**A**–**D**) Brightfield images of dorsal trunks of wildtype or *nacre/mitfa* mutant larvae at 2 dpf treated with 0.1% DMSO or 5 µM forskolin and processed for in situ hybridization using a *pnp4a* probe. A reduction in *pnp4a* is detected in wildtype (as previously observed) but not in *nacre/mitfa* larvae. Scale bar = 100 um and applies to all images. (**E**) Quantification of differentiated iridophores in 0.1% DMSO or 5 µM forskolin-treated *nacre/mitfa* larvae. We note a significant, yet attenuated reduction in iridophores with *mitfa* loss of function (22.2% reduction in iridophores or 77.8% of control). The reductions are significant via Student’s *t*-test (*p* < 0.05).

We next examined the effects of forskolin treatment on *pnp4a* expression in the dorsal trunk region of *nacre*/*mitfa* mutants. Again, treatment of wildtype embryos with forskolin caused a reduction in *pnp4a* expression at 2 dpf as compared to controls (Figure 5A,B; consistent with previous results in Figure 3). Conversely, *pnp4a* expression was similar in DMSO/control and forskolin-treated *nacre* larvae at 2 dpf (Figure 5C,D). To confirm that iridophore number was less impacted by PKA activity in *nacre* mutants with forskolin treatment, we quantified the number of iridophores in untreated and forskolin treated *mitfa*/*nacre* larvae at 4 dpf (following a washout at 2 dpf). While wildtype/AB larvae at 4 dpf showed a 38.4% (See Figure 1) reduction in iridophores as compared to DMSO treated controls, *nacre* larvae showed a 22.2% reduction in iridophores (Figure 5E). Furthermore, only the forskolin-treated *nacre* mutant iridophore number was close to control numbers by 7 dpf (control, 46.6 ± 4.4; forskolin 43.2 ± 4.8). Taken together, this data suggests that zebrafish larvae have a population of iridophores that are not sensitive to PKA in the absence of *mitfa* expression. 

### 3.4. Activation of PKA Expands Neural Crest Marker foxd3 Expression

**Figure 6:**
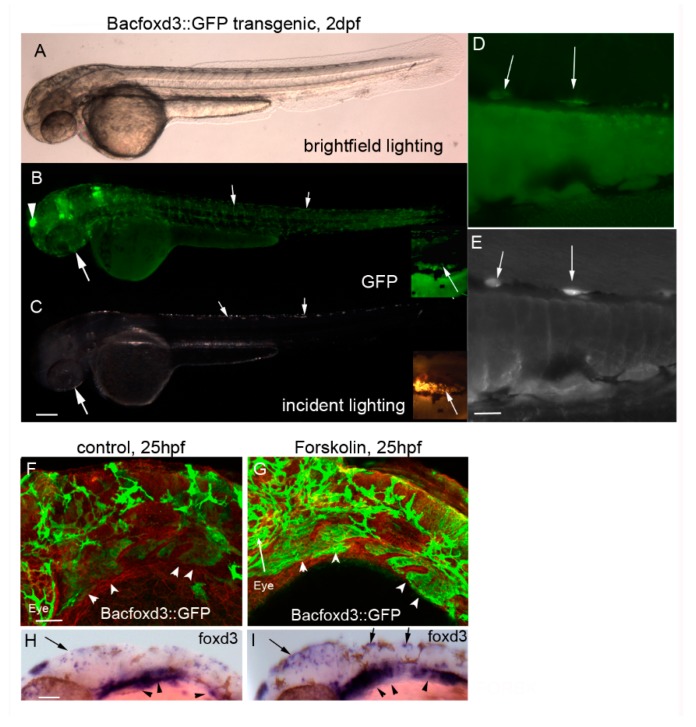
Forskolin increases expression of the *mitfa* transcriptional repressor *foxd3*. (**A**–**C**) 10× magnification of *Bacfoxd3::GFP* transgenic larvae at 2 dpf. Transgenic zebrafish express GFP in *foxd3*-dependent cells including the pineal gland (arrowhead) and presumptive iridophores (arrows). Scale bar in panel C = 200 um and applies to panels A–C. (**D**–**E**) 20× magnification of *BACfoxd3::GFP* zebrafish. Arrows indicate cells co-expressing GFP and iridescence typically observed in iridophores. The scale bar in panel E = 100 um and applies to panels D and E. (**F**,**G**) 20× magnification of 25 hpf 0.1% DMSO or 5 µM forskolin-treated *BACfoxd3::GFP* transgenic fish processed for immunocytochemistry using anti-GFP and anti-beta catenin antibodies (red). Forskolin treatment leads to an increase in GFP (arrowheads indicate pharyngeal arches), indicating expanded domains of *foxd3* expression. Scale bar in panel F = 100 um and applies to panels F and G. (**H**,**I**) 0.1% DMSO and 5 µM forskolin-treated wildtype embryos processed for in situ hybridization using the *foxd3* probe. Forskolin expands the areas of *foxd3* expression, notably in the dorsal head and trunk regions (arrows) which are locations of presumptive iridophores, and in pharyngeal arch neural crest streams (arrowheads). Scale bar in panel H = 100 um and applies to panels H and I.

Forkhead box transcriptional repressor *foxd3* activity is important for specification of specific neural crest derivatives, including iridophores [23,24] and melanophores [25]. *foxd3* is a well characterized marker for neural crest cells and presumptive iridophores. Consistently, *foxd3* is co-expressed with the iridophore marker *pnp4a* [10]. Additionally, evidence suggests that *foxd3* represses its own transcription [23,24]. *foxd3* intersects with several signaling pathways—including the ERBB3 signaling pathway in melanoma cells—via upregulation of specific signaling genes involved in cell proliferation and apoptosis [26,27] and directly represses *mitfa* expression—likely a necessary step in cells destined to become iridophores. Additionally, *foxd3* is required for *pnp4a* expression by developing iridophores [10]. We hypothesized that PKA inhibits *foxd3*, leading to increased *mitfa* expression/melanophore differentiation and decreased *pnp4a* expression/iridophore differentiation. To test this hypothesis, we used transgenic zebrafish expressing GFP under the control of the *foxd3* promoter (about 130 kb from BAC clone 137512) and conducted in situ hybridization using a probe against *foxd3*. Analysis of GFP transgenics at 3 dpf (Figure 6A–E) shows GFP expression in the expected *foxd3*-dependent locations, including the pineal gland (Figure 6B, arrowhead) and the skin (Figure 6B,C), with presumptive iridophores indicated by arrows [23,24,28]. Higher magnification (20×) confirms that GFP colocalizes with iridophores (Figure 6D–E). To determine the effects of forskolin treatment on *foxd3* expression, we treated embryos with forskolin or DMSO control embryo media from 19 to 25 hpf, fixed them in 4% PFA, and probed them using anti-GFP and anti-beta catenin antibodies. The analysis of beta catenin was added to better visualize cell boundaries. Unexpectedly, forskolin expanded the area(s) of *foxd3* expression as detected by the GFP reporter (Figure 6F–G; arrowheads indicate neural crest positive pharyngeal arches) and in situ hybridization (Figure 6H–I). Dorsal head and trunk skin also showed a wider domain of *foxd3* expression following forskolin treatment (arrows, Figure 6H–I). We also note similar locations of *foxd3* expression, eye size, and pharyngeal arch development in forskolin-treated embryos as compared to control treated embryos, suggesting that 5 μM forskolin has minimal impact on other developing tissues at this stage. Taken together, this data suggests that PKA does not inhibit iridophore differentiation by regulating *foxd3* expression. 

In summary, we have presented data supporting a role of PKA signaling in pigment cell differentiation. Specifically, we propose a model where PKA inhibits iridophore development while simultaneously promoting the development of a small subset of melanophores. PKA may also regulate melanin content as we noted changes in melanophore appearance and/or apparent melanin content with both treatments. Because the number of melanophores is so much greater than iridophores at the stages investigated, future studies will use a combination of qualitative and quantitative approaches to characterize the temporal and spatial requirements for PKA activity during iridophore and melanophore development. Although PKA does not inhibit *foxd3* expression, it is possible that *foxd3* protein activity is altered or that different mechanisms are at work in caudal versus trunk *foxd3*+ neural crest cells. Alternatively, PKA may activate *foxd3* in cells not destined to become iridophores or melanophores (i.e., glia, neurons or other *foxd3*-dependent cells). Future studies will investigate the fate of these PKA-activated *foxd3*+ cells as well as the localization and phosphorylation status of Foxd3 protein.

## 4. Conclusions

Specification and differentiation of neural crest cells is a highly regulated process, relying on expression (or inhibition) of specific transcription factors and cell differentiation genes in response to environmental signals. Our work suggests that protein kinase A signaling turns on melanophore specific programming at the expense of iridophore programming in a subset of chromatophore precursor cells. This is the first study to implicate PKA signaling as an intracellular regulator of iridophore development.
